# One-Stage Virtual Plan of a Complex Orthodontic/Prosthetic Dental Rehabilitation

**DOI:** 10.3390/ijerph19031474

**Published:** 2022-01-28

**Authors:** Alessandro Nota, Alisa Dmitrievna Chegodaeva, Alexander Nikolaevich Ryakhovsky, Maria Alexandrovna Vykhodtseva, Laura Pittari, Simona Tecco

**Affiliations:** 1 Dental School, Vita-Salute San Raffaele University, 20132 Milan, Italy; nota.alessandro@hsr.it (A.N.); laura_pittari@hotmail.it (L.P.); 2 National Medical Research Centre of Dentistry and Maxillofacial Surgery, 119021 Moscow, Russia; drchegodaevaalisa@gmail.com (A.D.C.); avantis2006@mail.ru (A.N.R.); dr.vihodzeva@mail.ru (M.A.V.)

**Keywords:** digital dentistry, computer-assisted planning, prosthetic rehabilitation, virtual patient, digital plan, orthodontic rehabilitation, lower jaw repositioning, centric relation, clear aligners, removable appliances, digital orthodontics

## Abstract

In complex dental treatments, a preliminary virtual plan (VP) can minimise the probability of errors and increase the predictability of the achieved result. Digital technologies and artificial intelligence open more opportunities for such planning, as they can be applied at the early stages of clinical examination to develop a simultaneous VP of all stages of treatment. The present clinical case describes a one-stage entire VP combining all the stages of treatment: gnathological, orthodontic, and prosthetic rehabilitation, until the final result. This approach avoids the accumulation errors associated with multistage VP, in which one stage of planning follows the end of a previous stage. One-step VP also allows demonstrating to the patients the expected results of the restoration, which increases their motivation to initiate the treatment.

## 1. Introduction

In complex dental treatments, early virtual planning is of great importance because it minimises the probability of errors and increases the predictability of the achieved result [1,2]. Digital technologies create more opportunities for such planning [3,4,5] because they can be applied at the early stages of initial examination to develop simultaneous planning of all stages of treatment until the final restoration [6,7,8,9].

The first step is to establish a mandibular position according to a correct intra-articular width in the two temporomandibular joints (TMJs). Several literature sources described methods that can be applied to establish this first step. For example, recently, a computer-aided method was developed to project mandibular repositioning using a Dental Cone-beam Computed Tomography (CBCT) [10]. First, the orthodontic models of enrolled subjects are digitised in StereoLithography (STL) format in maximum intercuspation. Patients then wear standardised 2 mm thick splints (mandibular repositioning) during CBCT imaging. On the CBCT, the mandible is virtually “separated” from the rest of the skull, and the STL files of the dental arches in maximum intercuspation are matched to the upper and lower jaw. The separated mandibular volume is then registered on the mandibular dentition using the teeth surfaces as a guide allowing the clinician to obtain two 3D images of condyles: one in habitual occlusion and the other after mandibular repositioning [10]. In this method, the mandibular repositioning is first decided clinically and only subsequently evaluated with CBCT. Therefore, although it may be considered a valid alternative to clinical repositioning, it may not be the best choice, as it still depends on prior clinical assessment. 3D software was recently introduced to assess and modify the position of the condyles, for example, by measuring intra-articular space widths (height) using CBCT images [11,12]. This software can also match intraoral scans with CBCT images, thus allowing virtual repositioning of the mandibular condyles based on the width of the intraarticular spaces. 

Nota et al. [6] described a fully digital method to design (Computer-Aided Design/Computer-Aided Manufacturing (CAD/CAM)) and mill a splint that can achieve the previously planned condylar position. This allows verifying whether the virtually planned condylar repositioning coincides with the position obtained wearing the occlusal splint designed and milled through this complete digital workflow. 

This case report presented here is based on a similar method. A recent review [13] reported numerous approaches to performing various steps involved in the virtual patient mounting, citing approximately 15 studies through PubMed on methodologies for mounting and analysing virtual patients. The review concluded that the digital methodologies involved in virtual patient mounting are not yet completely codified or standardised, and not one type of technology has not been shown superior to others. It also concluded that planning lower jaw repositioning has not yet been well studied, and additional research is needed in this respect [14]. The method described in the case study presented here will likely find indications in the analysis of intra-articular space width. 

However, as there are currently no published clinical reports describing a virtual plan of a complete dental rehabilitation, the present case study is the first to develop a virtual assembly showing lower jaw repositioning, orthodontic treatment, and subsequent prosthetic finalisation showing promising results for this diagnostic and treatment-planning tool. Thus, more precisely, the present case study describes a clinical example of one-stage digital planning and its implementation of the digital 3D technologies and software to perform automatic segmentation of temporomandibular joint (TMJ) for the purpose of an evaluation of TMJ intra-articular width. 

## 2. Materials and Methods

**Figure 1:**
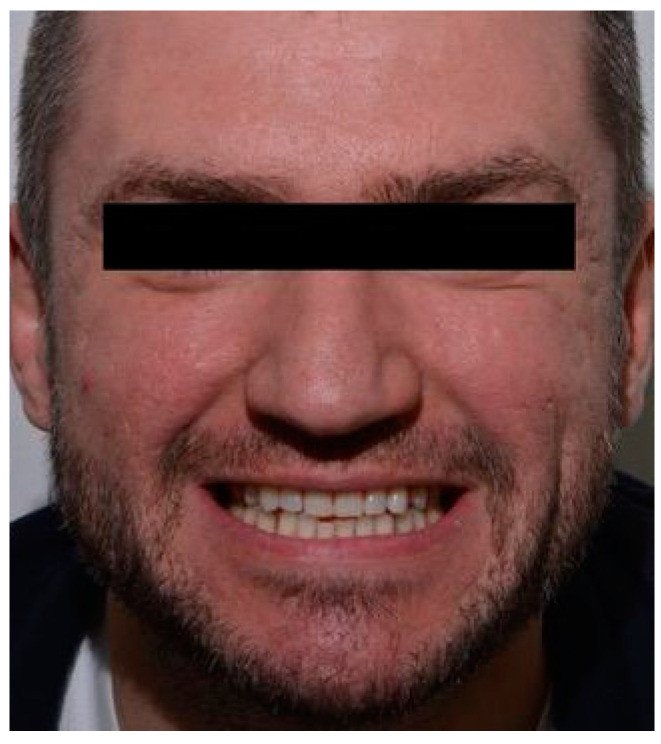
Initial extra-oral smile photo.

**Figure 2:**
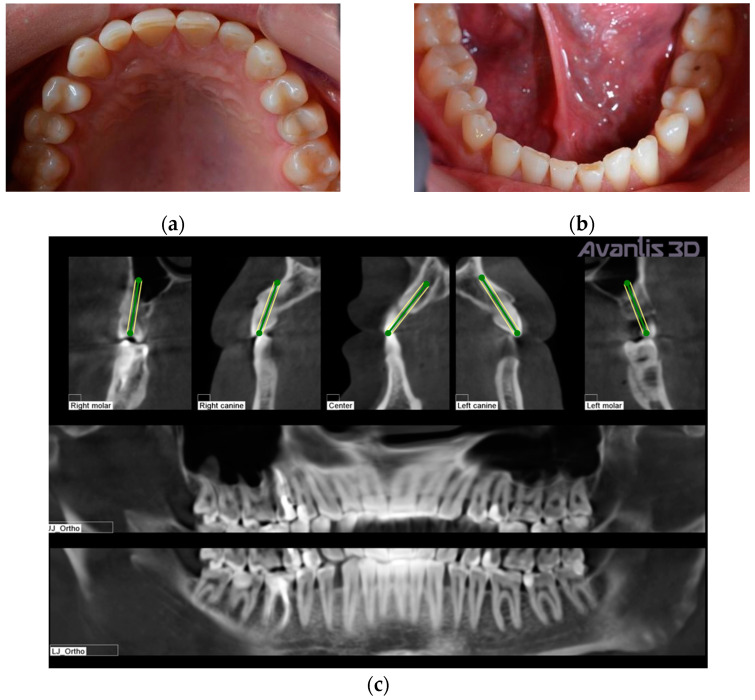
Intraoral occlusal views: (**a**) upper dental arch; (**b**) lower dental arch; (**c**) X-ray before treatment.

Initial clinical examination of an adult male patient (Figure 1) evidenced teeth wear, diastema between teeth, and small teeth. The patient did not complain of any discomfort around the temporomandibular joint and masticatory muscles. Previously, the patient was treated in another dental office for carious lesions, with composite restorations on teeth 17, 16, 15, 14, 22, 23, 24, 25, 26, 27, 37, 36, 35, 45, 46, 47. When we met the patient, generalised wear was evident on the occlusal surfaces (Figure 2). The first step of the treatment was professional oral hygiene. (Figure 2).

**Figure 3:**
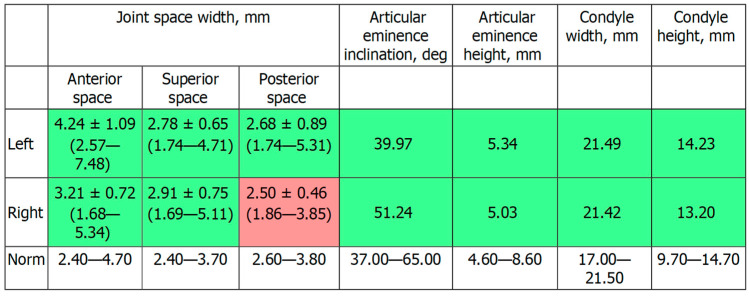
Table showed by the software. Intra-articular TMJ space widths before treatment. As represented in red, the posterior space width appears reduced with respect to normal values on the right TMJ.

Figure 2c shows the patient’s X-ray. Initially, digital technology was used to construct the “virtual patient”. Intraoral scan and CBCT reconstruction of facial area was obtained. The virtual patient was then analysed using Avantis3D software (LLC Avantis3D, Bolshoy Boulevard, 42/1, Off 137/22, Skolkovo. 121205 Moscow, Russia) and a one-stage virtual plan of the whole treatment was obtained. First, occlusal analysis confirmed tooth wear assessment of grade II (enamel and dentin) [15]. Then, the analysis of TMJs evidenced an asymmetry of the intra-articular space width of the right and left joints. There was a slight narrowing of the posterior third of the joint space on the right, while this same indicator was near the lower limit of the normal range on the left side [6,11,16,17], as represented in Figure 3.

**Figure 4:**
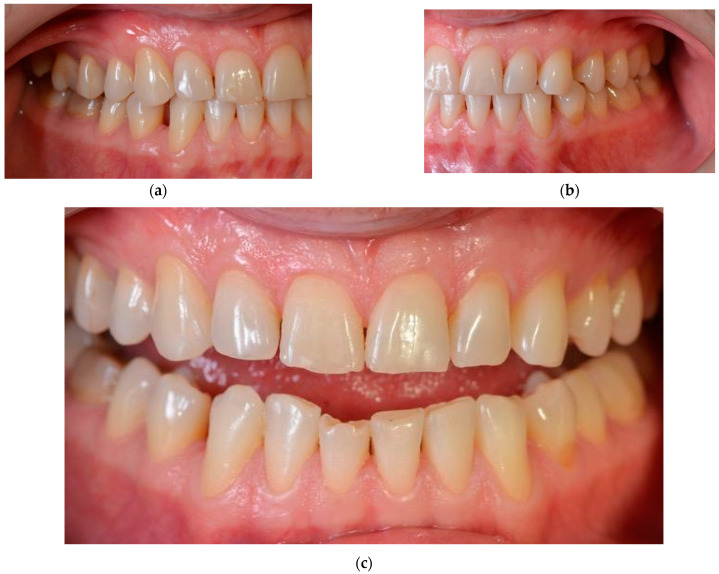
Intra-oral frontal and lateral views before treatment: (**a**) right lateral view; (**b**) left lateral view; (**c**) frontal view. Teeth appeal before treatment.

Intra-oral frontal and lateral views evidence diastemas between the canines and the first premolars and a slight crowding of the lower frontal teeth (Figure 4). 

The same software was used to perform a preliminary one-stage virtual planning to plan the whole treatment before beginning. 

It included the following steps: (a) the determination of occlusal vertical dimension through the analysis of lower jaw repositioning (LJ position); (b) the orthodontic correction of the position of the teeth to close diastemas and solve crowding; (c) the revision of the consistency of composite restorations before prosthetics; and (d) the manufacturing and fixation of ceramic restorations (veneers, inlays/onlays, and crowns). 

**Figure 5:**
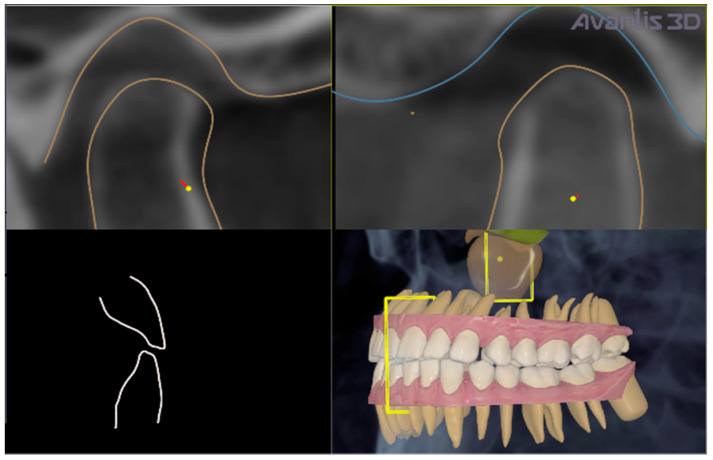
Virtual LJ repositioning. Repositioning of the right mandibular condyle consisted of its slight shift, forward and down, to normalise the intra-articular posterior space width of the right TMJ. It is traced in red colour.

**Figure 6:**
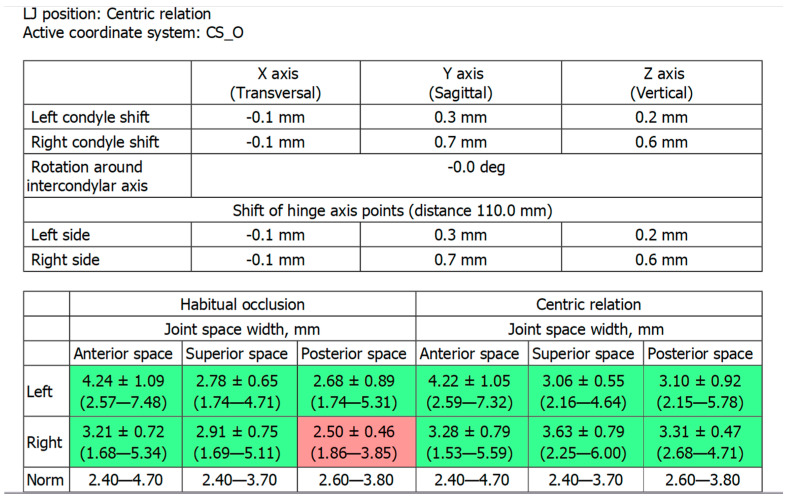
Table showed by the software. Intra-articular space widths in the right and left TMJs, before treatment (habitual occlusion, represented in the left part of the table) and in the one-stage virtual plan (centric relation, represented in the right part of the table). Posterior space width in the right TMJ passed from 2.5 mm to 3.31 mm, achieving a normal range. The upper part of the figure represents the mm of condylar repositioning. In the present case, the right condyle was moved 0.7 mm forward and 0.6 mm down.

Firstly, LJ position was virtually determined; in the present clinical case, LJ repositioning consisted of a slight shift of the LJ, forward and down, to obtain a normalisation of the intra-articular posterior space width of right TMJ, without rotation of the LJ around its anatomical hinge axis to increase the occlusal vertical dimension (Figure 5 and Figure 6). 

After planning, the patient was treated with a professional hygiene procedure to obtain general decontamination and reduce the number of bacteria in the mouth. Endodontics procedures were not performed because the patient arrived at the dental clinic with already treated 1.5 and 4.6 teeth. These treatments were not repeated because the teeth did not show any other endodontic problem. 

**Figure 7:**
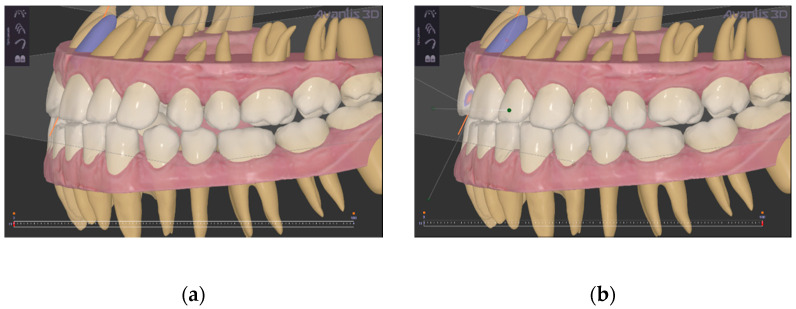
Virtual teeth alignment. (**a**) before virtual alignment; (**b**) after virtual alignment.

After this step, the alignment of teeth was planned with the same software using the virtual repositioning as an initial condition of the orthodontic treatment to close diastemas between the teeth and adapt teeth to the planned mandibular repositioning (the operator in the software performed all corrections of malpositions) (Figure 7). Each aligner was moulded in polyurethane material on the dental models to achieve alignment. Each movement was about 0.25 mm/aligner. The width of the polyurethane disk is 1 mm (Pro-Form, Keystone, CO, USA). 

**Figure 8:**
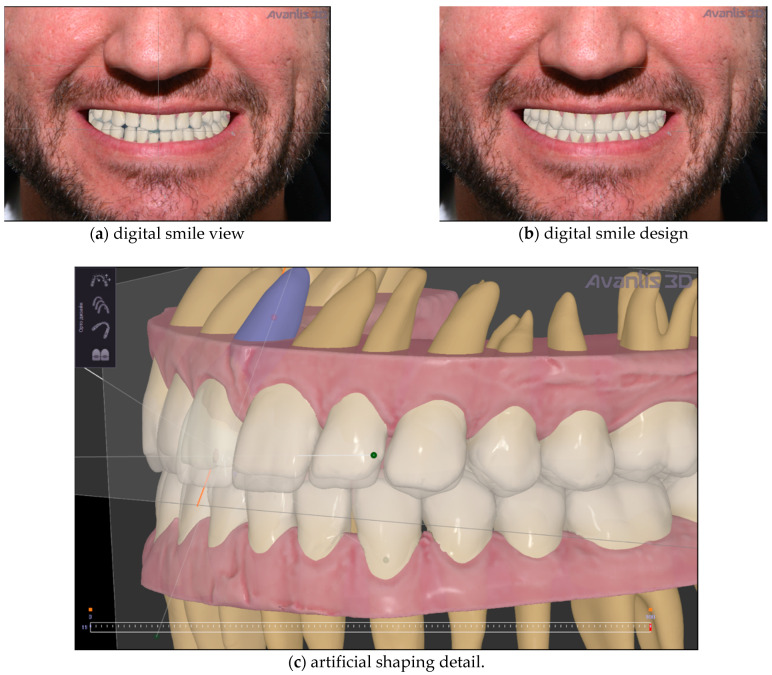
Artificial teeth setup. (**a**) digital smile view; (**b**) digital smile design (**c**) artificial shaping.

Then, artificial teeth were set up, from the final position of orthodontic treatment, to improve teeth shape and ultimately establish the planned vertical dimension (Figure 8).

**Figure 9:**
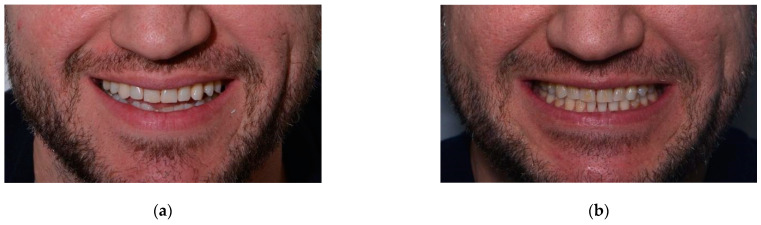
Intra-oral smile view before (**a**) and after (**b**) teeth alignment.

After one-stage virtual planning, the patient was invited to see his new smile simulation (Figure 8). He was completely satisfied with what he saw and agreed to the treatment. So, from a clinical point of view, the orthodontic correction was initially performed using clear aligners (Figure 9). This treatment took five months. It used NexDent 5100 (3D Systems) to print models for Aligners (material–Model Ortho), printing all models to make thermoformed aligners. The patient changes aligners by himself every 14 days and make control visits (one in the middle of the treatment and a few at the end to make an approximal reduction of the teeth, if necessary).

**Figure 10:**
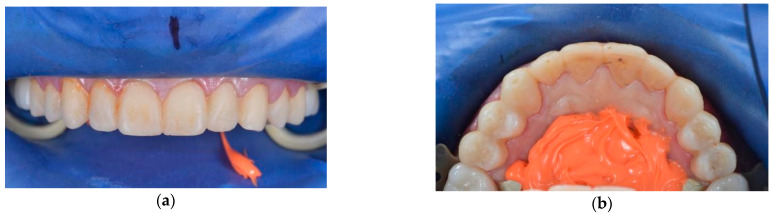
Frontal (**a**) and occlusal (**b**) view of teeth mock-up.

Due to the simplicity of the initial clinical situation, the patient was given the entire set of clear aligners, and he consistently changed them independently, appearing only one time in the middle of treatment for a control examination. At the end of orthodontic treatment, based on the previously planned LJ position and design of artificial teeth, a mock-up model was printed, and teeth shape was transferred to the oral cavity through a silicone paste, creating temporary restorations (Figure 10).

The mock up was created using silicon indexes and Luxatemp (DMG) material for temporaries and Luxaflow (DMG) for bonding. The patient wore the mock-up and accepted the planned shape of teeth.

**Figure 11:**
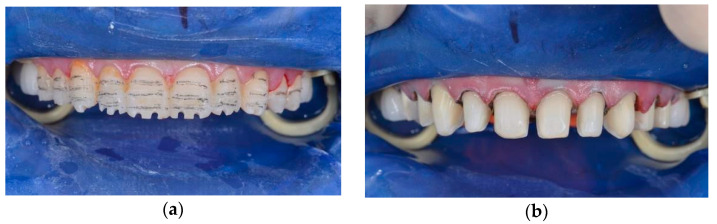
Teeth preparation (**a**,**b**).

**Figure 12:**
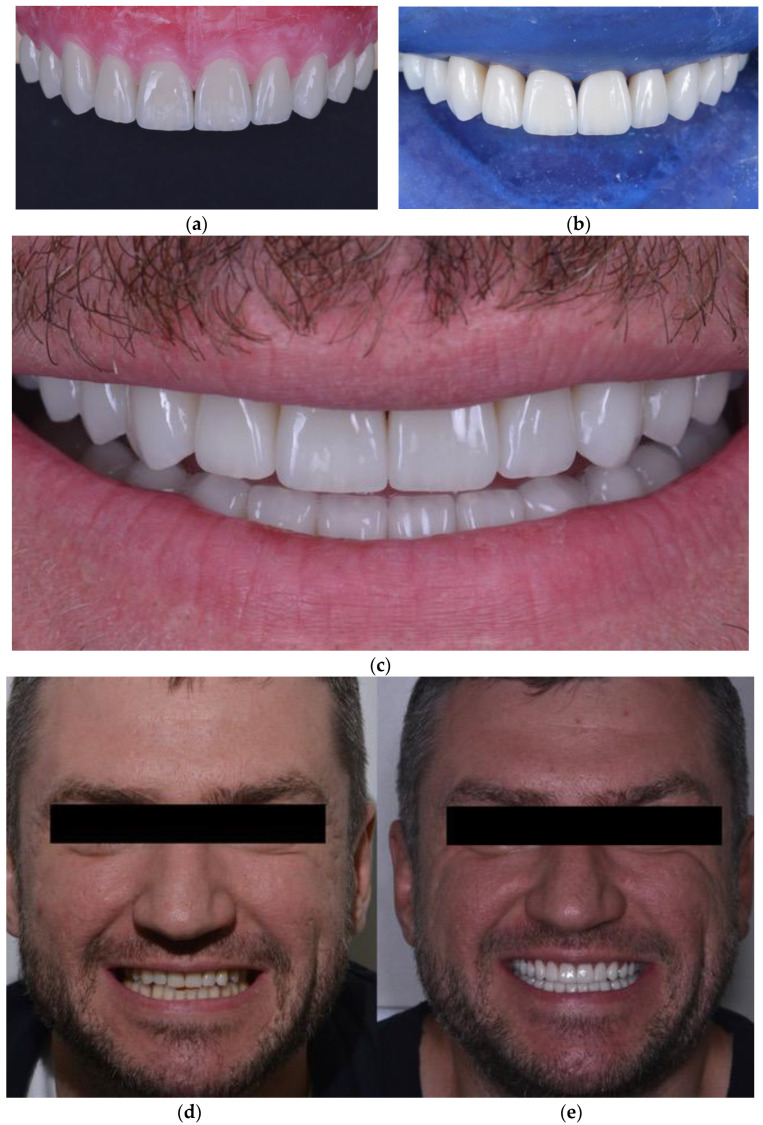
Ceramic restorations fixed on the teeth (**a**–**c**); smile view before (**d**) and after (**e**) treatment.

The mock-up changed not only the shape of the teeth but also the occlusal vertical dimension. Assessing the clinical acceptance of treatment, the prosthodontist performed the teeth preparation (Figure 11). Then, the manufacture of prosthetic dentures was performed, giving group guidance to prevent overloading. Ceramic prosthetics were manufactured after performing a new intraoral scan using the Emax technology (under isolation with the Rubber Dam), followed by individualising shape and colour by manually applying the lithium disilicate ceramic mass to the vestibular surface of indirect restorations. The technician designed onlays and veneers in Exocad. Then, onlays and veneers were milled from the wax first (Vhf), and then eMax was pressed. All frontal teeth were vital; preparation was conducted using a silicone index, so all frontal teeth had a similar colour. Restorations were tried on and fixed on the teeth with Variolink Esthetic LC (neutral) light-curing cement (Figure 12).

At the end of treatment, the patient was followed to evaluate the periodontal status of the gingiva, and the oral hygiene status. The general oral and periodontal conditions were acceptable, and the patient did not manifest any periodontal problems all over the last year follow-up. Control and minor occlusal corrections were made after bonding, after one week, one month and three months, performed with a handpiece and diamond buyers with different abrasiveness for ceramics (DZ ceramic Polishing Kit).

## 3. Results

**Figure 13:**
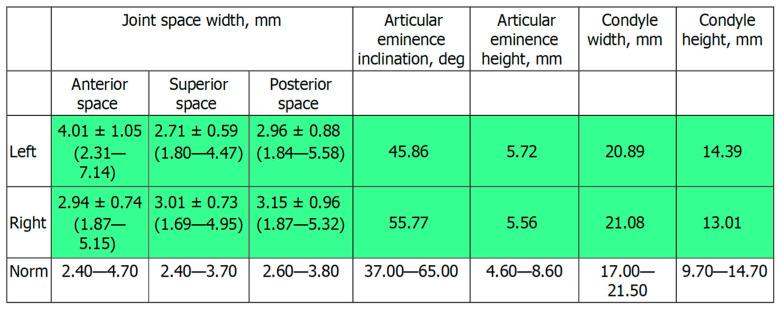
Table showed by the software. TMJ analysis after dental treatment, with normal intra-articular space widths both in the left and right TMJ.

At the end of treatment, the patient was completely satisfied with the functional and aesthetic results of the treatment. A control CBCT scan was performed one year after the end of treatment and confirmed the correctness LJ position, as TMJ analysis showed normal intra-articular space widths (Figure 13).

## 4. Discussion

The present clinical case shows one-stage virtual planning of a complex treatment involving three different types of interventions that were planned at once: gnathological, orthodontic and prosthetic. 

In the present case, the same software (Avantis3D) was used to perform the whole virtual planning. 

To the best of the author’s knowledge, there are no other commercially available systems for whole virtual planning regarding the modalities of integrating radiographic data, virtual dental models, and the possibility of analysing TMJ and intra-articular space width with lower jaw repositioning. 

A method has been recently proposed in the Japanese language for determining the jaw position of repositioning splint involving CBCT of TMJSs and intraoral scans. However, the choice of the mandibular position is made on the base of the distance between teeth in the anterior dental arches and not on the base of TMJ intra-articular space width, as no TMJ analysis is performed [18].

Differently, the present system has a “Digital Imaging and Communication in Medicine” DICOM interface to import radiographic data. The import of virtual dental models in a universal format (STL: Standard Tesselation Language) is possible, and the system displays three-dimensional surface models or two-dimensional cross-sections with varying orientations for virtual planning. Computer-aided design and manufacturing (CAD/CAM) of the orthodontic and prosthetic appliances may be performed by the user with the help of default parameters or solely by the provider of the software and thus without the influence of the clinician.

Regarding the phase of orthodontic planning, the present system allowed using the teeth position achieved after condylar repositioning (Figure 5) as the initial position for digital planning of orthodontics (Figure 7a). Conventionally, the planning of orthodontic treatment in patients previously treated with oral appliances for the temporomandibular joint disease involves using temporary occlusal composite bite blocks necessary to increase the vertical dimension to allow teeth extrusion [19]. However, the present system has overcome this necessity, allowing a complete one-stage digital planning.

Regarding the phase of smile design, the present protocol allowed the merging of smile design to recognise digitally where to position the teeth using orthodontic movement. This approach allows the desired esthetic design to be attained while achieving a better position of teeth and spaces distribution, allowing minimisation of the dental waste needed to perform ceramic restorations. Figure 9a,b evidence the results after clear aligner treatment and evidence lack undesired spaces between upper and lower teeth. This is a rationale approach already evidenced in literature [20].

As seen, the present case study outlines a comprehensive interdisciplinary virtual plan for developing functional and esthetics rehabilitation. The case illustrates the utility of a whole virtual plan for an interdisciplinary treatment in which specialists are recruited on the same plan to discuss their therapeutical proposals.

A recent review [7] reports that the digital methodologies involved in virtual patient mounting are not yet completely codified or standardised, and one type of technology has not been proven superior to others. It also concluded that planning of lower jaw repositioning has not yet been well studied, and additional research is needed in this respect [8]. Thus, the method described in the present case study will likely find indications in intra-articular space width analysis.

Limitations of the described system concern the necessity of a preliminary CBCT acquisition, the lack of a real cinematic acquisition of the patient’s mandibular movements, and the lack of TMJ’s disc visualisation. Another limitation of this method could be the fact that data about the function of the muscles (for example, the electromyographic potentials at rest and during clenching [21]) must be analysed separately and cannot be integrated into the overall evaluation. The visualisation of the articular disc could allow programming the new LJ position more accurately, keeping in mind the physio-pathology of the disc-condyle complex. In the future, this type of visualisation could be allowed by matching disk images extracted from an MRI, for example. While as regards the acquisition of data on mandibular kinematics, they could be extracted and integrated into the system using a preliminary kinesiographic survey.

Moreover, in the present case study, it was not possible to perform a post-treatment CBCT to assess the final LJ repositioning to limit the patient’s X-ray exposure according to the ALADA principle. A previous study [6] performed with the same software for milling an occlusal splint with a complete digital workflow showed that the digital planning accurately achieved the previously planned condylar position and intra-articular spaces.

One-stage virtual planning avoids the “accumulation” errors associated with multistage virtual planning, in which each step of virtual planning follows the end of the previous step of clinical treatment. Accumulation error is the overall effect of rounding-off at the various stages of a procedure on the accuracy of the final solution: several very slight errors that are not significant in the context of a particular step, if added together, can affect the accuracy of the final result.

## 5. Conclusions

The present clinical case describes a one-stage whole VP involving all the stages of treatment, combined at once: gnathological, orthodontic and prosthetic rehabilitation, until the final smile view.

## Data Availability

The data presented in this study are available on request from the corresponding author. The data are not publicly available due to privacy.
